# The Economic Burden of Breast Cancer Survivors in Korea: A Descriptive Study Using a 26-Month Micro-Costing Cohort Approach

**DOI:** 10.31557/APJCP.2019.20.7.2131

**Published:** 2019

**Authors:** Chang Hoon You, Sungwook Kang, Young Dae Kwon

**Affiliations:** 1 *Graduate School of Public Health, Yonsei University, 50-1 Yonsei-ro, Seodaemun-gu,,*; 3 *Department of Humanities and Social Medicine, College of Medicine and Catholic Institute for Healthcare Management, The Catholic University of Korea, 222 Banpo-daero, Seocho-gu, Seoul,*; 2 *Department of Public Health, Daegu Haany University, 1 Haanydaero, Gyeongsan, Korea. *

**Keywords:** Breast cancer- out-of-pocket expenses- health insurance- cancer burden- survivors

## Abstract

**Background:**

This study analyzed the burden of cancer treatment costs on patients by calculating the monthly amount of medical expenses paid by breast cancer patients for two years after mastectomy.

**Methods:**

Among those who were diagnosed with breast cancer and had received treatment at one of two academic medical centers in Seoul between 2003 and 2011, 1,087 patients who underwent mastectomy and received follow-up for at least two years were recruited. A micro-costing approach from the provider’s perspective, based on a retrospective review of patient medical claim records, was used to analyze cancer treatment cost of care. The cohort’s number of hospitalizations, total hospitalization duration, and number of outpatient visits were noted, and the total amount of medical expenses, out-of-pocket (OOP) expenditures, uninsured costs, and OOP ratio were calculated.

**Results:**

The total amount of medical expenses tended to increase by year, whereas the OOP expenditure ratio decreased. The OOP expenditure ratio was highest in the first month post-operation. Around one quarter of the total OOP payments incurred over the course of three months: one month before the operation, the month of the operation, and one month post-operation.

**Conclusion:**

OOP payment burden on patients was concentrated in the initial phase of treatment, and items not covered by the National Health Insurance caused an additional increase in patients’ burden in the initial phase. The economic burden of cancer treatment varies considerably. In order to alleviate patients’ medical expenses burden, the timing of expenditures and the possible financial burden on cancer survivors, they should be understood more fully and possibly addressed in interventions aimed at reducing the cancer burden.

## Introduction

Cancer is one of the most common serious diseases in South Korea, being the leading cause of death for both men and women since 2000. But due to continuous early diagnosis and prophylactic measures, the standardized incidence ratio of cancer has been on the decline, decreasing from 324.9 per 100 thousand people in 2011 to 289.1 per 100 thousand people in 2014. As a result of increased early treatment and advanced treatment technologies, cancer survival rates are also rising. The five-year relative survival rate of cancer patients from 2010 to 2014 was 70.3%, exceeding the 70% line for the first time. 

Due to the increase in life expectancy after cancer occurrence and active introduction of new medical technologies in diagnosis and treatment, the costs for treatment and the overall social burden from cancer is also continuously (Warren et al., 2008; You et al., 2013). Cancer accounts for 13-17% of the global burden of disease, and 4-7% of all medical expenses. In the United States, cancer comprises approximately 5% of gross medical expenses, and cancer treatment costs are expected to rise 40%—from $125 billion in 2010 to $175 billion in 2020 (Mariotto et al., 2011). South Korea is showing a similar trend, where cancer treatment costs accounted for 4.9% of the total payment made by the National Health Insurance Service (NHIS) in 2001, but in 2011 this amount increased to 8.2%.

Until now, most studies on cancer treatment costs focused on estimating the total amount along with a detailed analysis based on the type of cancer, treatment method, stage of illness, survival period, and socio-demographic characteristics. Recent studies have analyzed the medical expenditure pattern from the moment of diagnosis to the moment of death (or other specific time), focusing on treatment cost burden for patients. Results of many studies show that the expenditure pattern starting at cancer diagnosis or the start of treatment forms a U-shaped curve, with the greatest amount of medical expenditures falling within the first year after diagnosis (Brown et al., 2002; Meropol et al., 2007; Yabroff et al., 2007; Yabroff et al., 2008). Generally, cancer patients receive intensive treatment in the short time period right after the diagnosis, which puts a significant financial burden on patients due to the high medical expenditure (Langa et al., 2004; Wagner and Lacey, 2004; Meropol et al., 2009). The financial burden of treatment is also called financial toxicity, and is known to affect not only the mental well-being of a person but also decision-making about the appropriate treatment (Meropol et al., 2009). Because cancer treatment induces high treatment costs, the financial burden on patients is a crucial social issue and an important matter for the health insurance system (Zafar et al., 2013; Bestvina et al., 2014). 

Cancer patients’ perception of the burden of medical costs can differ from that of insurers in terms of time. Because insurers manage insurance benefits or premiums from an aggregate viewpoint on a macro level, it is generally accepted they approach the burden of medical costs for cancer patients on a long-term basis that spans the entire treatment period or by year. Related studies also tend to be carried out in similar ways, for example, analyzing medical expenditures from the point of cancer diagnosis to death; trends in medical expenditure incurrence at the initial, follow-up, and terminal stages; and semiannual or annual trends in medical expenditure incurrence (Riley et al., 1995; Kim et al., 2009). However, from the cancer patients’ standpoint, medical expenditure and its burden should be assessed in accordance with the household’s income, so expenditures should be assessed on a weekly or monthly basis. Several previous studies analyzed the development of cancer treatment cost incurrence by short time periods in this fashion (Riley et al., 1995; Yabroff et al., 2004; Cheong et al., 2013), but there have not yet been studies that analyze the aspect of the incurrence of out-of-pocket expenditures for cancer treatment on a short-term basis that spans less than a month. Thus, this study aimed to analyze the level of burden of treatment costs on patients by calculating the monthly amount of medical expenses paid by breast cancer patients for two years starting at their mastectomy.

## Materials and Methods

*Subjects and data*

A micro-costing approach from the provider’s perspective, based on detailed cost information for each patient at each admission or visit, was used in the analysis. This method analyzed the monthly incurrence of medical expenses of breast cancer patients for two years from the day they underwent mastectomy. Medical expenses for breast cancer patients include not only the cost of breast cancer surgery but also the cost of chemotherapy and radiation treatment. Among those who were diagnosed with breast cancer (International Classification Diseases-10 code C50) and had received treatment at one of two academic medical centers in Seoul between 2003 and 2011, 1,087 patients who underwent mastectomy and received follow-up were assessed. Both hospitals from which the study participants were selected were general hospitals with 1,332 and 570 beds each, and were tertiary medical institutions in the healthcare delivery system. Both hospitals were top-ranked with a nationwide reputation in cancer treatment. 

Patients who underwent reoperation within two years after the first operation due to recurrence or other causes were excluded from the study. However, those who had less than three months between their first and second operation were included in the study because the second operation may be performed depending on the results of radiological examination or biopsy for metastasis of the surgical site after partial mastectomy. Due to the difficulty of determining the time of diagnosis of the study participants, the starting point of the medical expenditure study period was set as the day that the patient underwent mastectomy. The study period included one month before the operation date, considering the preparatory process including preliminary examinations before the operation. Because the treatment expenses study period was set as two years from the time of the operation, only the cases where there was a confirmed record of visit and treatment under the same diagnosis within at least two years after the operation were included. Accordingly, cases where the patient died within two years after the operation, changed hospitals, etc., were excluded from the study.

The analyzed data were extracted from the hospital cost analysis system based on the corresponding hospitals’ medical bills, excluding the patients’ personal information. These are administrative data constructed with the purpose of charging patients medical costs, and contain detailed information on the type of service and its amount, time period, and costs, but do not offer much clinical information. Data used in this study were collected from only two hospitals, so the results are limited in terms of national representativeness. However, the data have the strong advantage of accuracy and reliability. This study was approved by the Institutional Review Board of The Catholic University of Korea (CUMC11U135) with a waiver for informed consent because the data were obtained from an administrative database and were analyzed anonymously.

*Statistical analysis*

Yearly cohorts were drawn by the time of operation. Using medical expense data from 2003 to 2011, we rebuilt six yearly breast cancer cohorts (2003-2008) that included all medical expenses for two years after breast cancer surgery at the hospital. Total medical costs were defined as the aggregate amount of medical expenditure incurred during the two-year period after the mastectomy (25 months in actuality, including the one month before the operation) in which the patient received treatments at the same hospital, and included all cases where the bills had C50 as the diagnosis. It included costs for outpatient, inpatient, and emergency treatment. 

South Korea implements National Health Insurance (NHI) that provides for all of its citizens. A diagnosis related groups-based payment system is applied to some patient groups, but in most cases, patients have their medical expenses calculated through the fee-for-service method. Service items covered by the NHI can be divided into the insurer’s payment and copayment by the patient (statutory copayment), and expenses for items not covered by the NHI are paid entirely by the patient (uninsured costs). Insured and uninsured services are determined based on medical validity and needs, with consideration of the NHI finances. Thus, some medically necessary services are included in uninsured items. Out-of-pocket (OOP) expenditures include copayments and uninsured costs, and the OOP ratio refers to the ratio of OOP expenditures to the total amount of medical expenses. 

The average and standard deviation for each yearly cohort’s number of hospitalizations during two years, total hospitalization duration, number of outpatient visits, total amount of medical expenses, OOP expenditure, uninsured costs, and OOP ratio were calculated. In order to determine at which point patient OOP ratio rose or fell, the OOP ratio value by month for two years was calculated and shown in a graph. In order to determine the size of the burden of medical expenses on patients by time during the study period, the ratio of the patients’ OOP payment to the total amount of medical expenses by month during two years were calculated and illustrated in a graph. This was divided into copayment costs and uninsured costs, the monthly ratio of which were each calculated and compared in a graph. In calculating the monthly values and showing their trends in a graph, cohorts were merged into three groups, combining two consecutive cohorts into one group each: 2003-2004, 2005-2006, and 2007-2008. In the graphing case, the six yearly cohorts were re-categorized into three to reduce complexity due to multiple trend lines and to reflect the lowering of the statutory copayment ratio from 20% to 10% in 2005. 

In order to reflect the inflation rate during the study period, each year was given a weight based on deflator calculated using Korea Consumer Price Index. We multiplied the total medical expenses by the deflator factors of each year. The annual average exchange rate of 2010 (1 US dollar=1,134.8 Korean won) was applied to convert the currency from South Korean won to US dollars. For all statistical analyses in this study, SAS ver. 9.2 (SAS Institute, Cary, NC, USA) was used.

**Table 1:** Medical Utilization and Expenditures from Breast Cancer Treatment in the Two Years after Surgery

Year of surgery	N	Total medical cost (USD)	Covered cost (USD)		Insurance not covered cost (USD)	Number of admissions	Length of stay (days)	Number of physician visits
			Insurance payment	Patient payment				
2003	105	9,473 ± 5,996	4,205 ± 2,662	1,031 ± 652	4,237 ± 2,682	2.0 ± 2.5	14.9 ± 22.7	21.2 ± 11.3
2004	192	11,817 ± 8,638	5,731 ± 4,189	1,315 ± 4,189	4,770 ± 3,487	2.9 ± 3.4	18.8 ± 20.6	23.5 ± 15.3
2005	201	11,450 ± 7,939	5,865 ± 4,066	967 ± 670	4,618 ± 3,202	2.6 ± 3.7	15.0 ± 15.8	24.3 ± 15.2
2006	188	12,442 ± 8,718	7,648 ± 5,359	850 ± 595	3,944 ± 2,764	2.9 ± 3.5	17.7 ± 21.3	23.5 ± 15.1
2007	203	13,796 ± 11,298	9,159 ± 7,501	1,014 ± 830	3,624 ± 2,967	3.5 ± 4.8	17.7 ± 20.4	27.5 ± 20.9
2008	198	13,670 ± 10,487	8,326 ± 6,388	844 ± 647	4,500 ± 3,452	2.2 ± 3.2	10.7 ± 17.5	30.1 ± 25.1
Total (mean ± SD)	181 ± 37	12,108 ± 8,846	6,822 ± 5,027	1,003 ± 726	4,282 ± 3,092	2.7 ± 3.5	15.8 ± 19.7	25.0 ± 17.2

USD, United States dollar; SD, standard deviation

**Figure 1:**
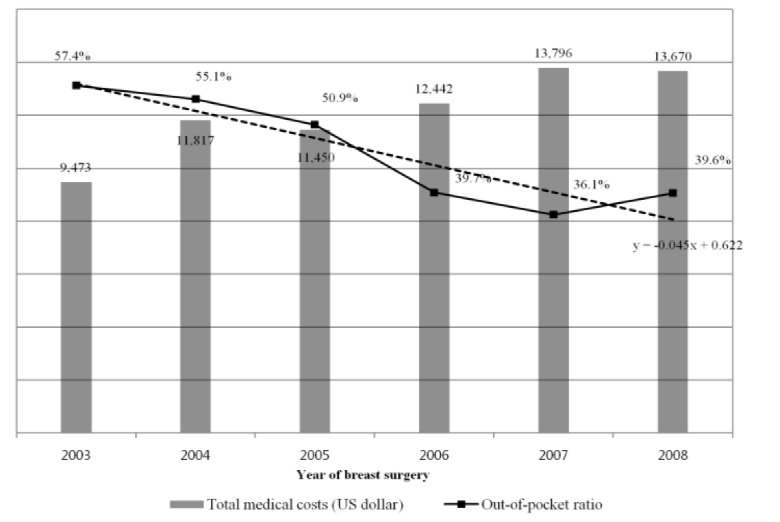
Total Medical Expenditure and Out of Pocket Ratio for Breast Cancer Patients in the Two Years after Surgery

**Figure 2:**
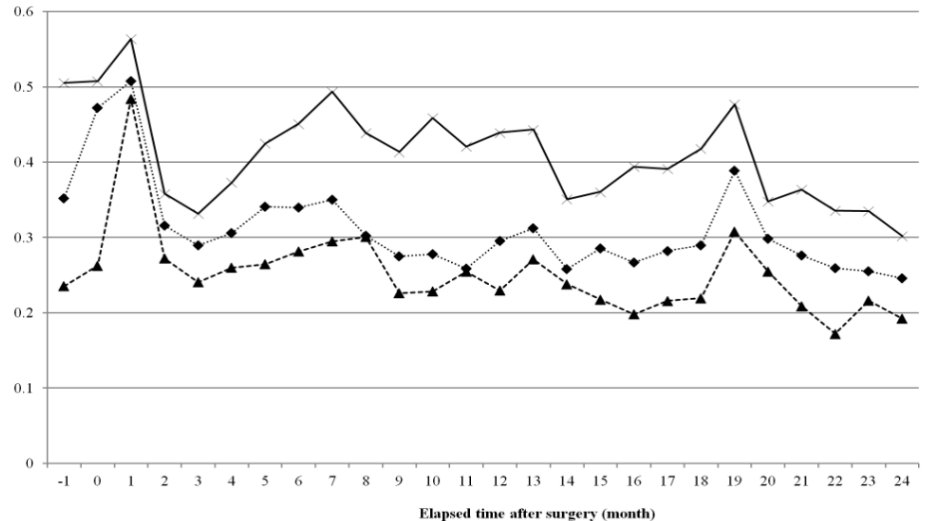
Trends of Out of Pocket Ratio due to Breast Cancer Treatment for Two Years after Surgery (Out of Pocket Ratio is Out of Pocket Expenses Divided by Total Medical Expenses at Each Month)

**Figure 3:**
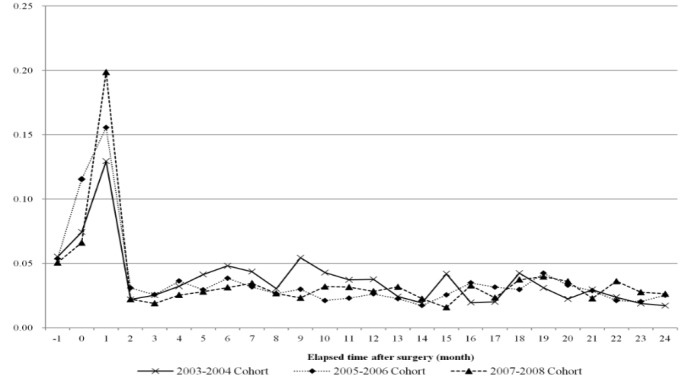
Trends of Monthly Proportion of Out-of-Pocket Expenditure Due To Breast Cancer Treatment for Two Years after Surgery (Monthly Proportion is Out-of-Pocket Expense at Each Month Divided by Two-Year Total Out-of-Pocket Expenses)

**Figure 4:**
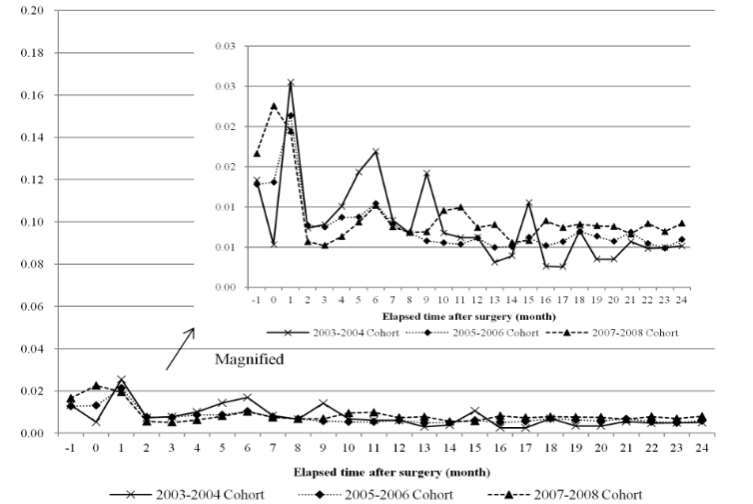
Trends of Monthly Proportion of Copayment Out-of-Pocket Expenditure Due to Breast Cancer Treatment for Two Years after Surgery (Monthly Proportion of Copayment Out-of-Pocket Expenditure is Copayment Out-of-Pocket Expenditure at each Month Divided by Two-Year Total Out-of-Pocket Expenses)

**Figure 5:**
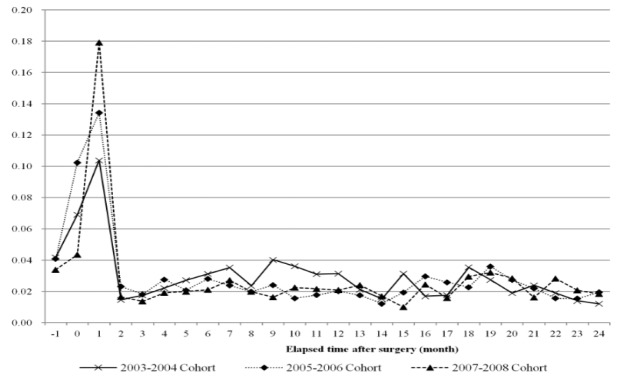
Trends of Monthly Proportion of Uninsured Out-of-Pocket Expenditure Due to Breast Cancer Treatment for Two Years after Surgery (Monthly Proportion of Uninsured Out-of-Pocket Expenditure is Uninsured Out-of-Pocket Expenditure at Each Month Divided by Two-Year Total Out-of-Pocket Expenses)

## Results

Table 1 presents the amount of medical expenses and medical service utilization for two years after patient mastectomy. Each yearly cohort included 105-203 study subjects. The total amount of medical expenses for two years after mastectomy ranged between $9,473 (2003) and $13,796 (2007), averaging $12,108, and showed an increase every year. The ratio of costs paid by the insurer (NHIS) to the total amount of the medical bills also increased, but patient copayment costs and uninsured costs did not show a regular trend, either increasing or decreasing, depending on the year. The average number of hospitalizations over the two years was 2.7 times, and the average duration of hospitalization was 15.8 days. The number of outpatient visits averaged 25.0, and showed growth by time, increasing from 21.2 times in 2003 to 30.1 times in 2008 (Table 1). 

Figure 1 shows the total medical expenditure and the OOP expenditure ratio (the ratio of copayments and uninsured costs to the total amount of medical expenditures) over the two years by yearly cohorts. The total medical expenditure of a patient who had a mastectomy in 2003 was $9,473 and the OOP expenditure ratio was 57.4%, whereas the total medical expenditure of a patient who had a mastectomy in 2008 was $13,670 and the OOP expenditure ratio was 39.6%. The total medical expenditures tended to increase by year, whereas the OOP expenditure ratio decreased until 2008, when it started to increase (Figure 1). 

Figure 2 illustrates the results of the analysis of monthly OOP expenditure ratio after mastectomy. In three cohort groups the OOP expenditure ratio was approximately 50% and highest in the first month post-operation; over time the OOP expenditure ratio started to decrease. In the 19th month post-mastectomy, the OOP expenditure ratio showed a distinct increase (Figure 2).

Figure 3 shows the amount of medical expenses paid monthly by the patient in relation to the total amount of the patient’s OOP expenditure over two years. This shows the time or period over which the burden of medical expenditure on patients was concentrated. All three cohorts showed a very similar trend where the first month after the operation accounted for the largest amount of OOP payment-15%. Approximately one quarter of the total OOP payments were incurred over the course of three months-one month before the operation, the month of the operation, and one month post-operation; this shows that there is a high burden of medical expenses in the early stages of treatment both before and after the operation. Out of the three cohorts, the more recent cohorts showed a higher concentration of medical expenses during the initial phase. From the second month post-operation, the ratio was under 5% and did not show much difference between groups (Figure 3).

Figures 4 and 5 each demonstrate the distribution of the ratio of patient monthly expenditure to the total OOP expenditures amount for two years, dividing patient expenditures into copayment and uninsured costs. Copayment costs were also high right before and after the operation, but were very low on the whole-approximately 2% and showed a tendency to decrease in more recent cohort groups (Figure 4). Uninsured costs were also the highest at the period of operation, but were much higher compared to copayment costs, exceeding 10%. More recent cohorts showed a higher ratio at the time of operation, and from the second month after the operation all groups showed an evenly low ratio of 4% or lower (Figure 5). 

## Discussion

This study is a descriptive study that tracked 1,087 breast cancer patients who underwent mastectomy at one of two academic medical centers in Seoul from one month prior to the operation to two years post-operation and analyzed medical expenditure incurrence trends. Analysis of total medical expenditures showed that over two years those who had surgery in 2003 paid $9,473, whereas those who had surgery in 2008 paid $13,670. In the analysis of medical utilizations, these results showed that the number of hospitalization and length of stay were similar, but the number of outpatient visits increased. As a result, the total medical expenses increased. It was analyzed that the increased medical expenses were borne mainly by the insurer rather than by patients.

In other words, over five years the total amount of medical expenditures increased by 44%. However, the amount of the patient’s OOP expenditure decreased by 12.0%, from $5,268 for patients who had surgery in 2003 to $4,637 for patients who had surgery in 2007 (increasing to $5,344 in 2008). As a result, patient OOP ratio showed a significant reduction from 57.4% among the 2003 cohort to 36.1% among the 2007 cohort (slightly increasing to 39.6% in 2008). 

Thus, total medical expenditures over the course of two years post operation increased with time, but the amount of OOP payments decreased; this is similar to the results of previous studies (You et al., 2013). This can be explained by the introduction of the OOP payment cap system by NHI in 2004 to lessen the burden of medical expenses on patients, and the consequent lowering of the statutory copayment ratio from 20% to 10% in 2005, and to 5% in 2009. NHI’s continuous efforts to extend the range of insured items also appear to have contributed. However, in the case of patients who had surgery in 2008, both the amount of the OOP payments and the OOP ratio increased due to an increase in uninsured costs. Despite the extension of the range of items covered by the NHI, active introduction of new medical technology including anti-carcinogens, robot therapy, diagnosis, and follow-up examinations may have contributed to the increase in uninsured costs (Cheong et al., 2013; . Jeon et al., 2015). 

Monthly OOP trends showed that the OOP ratio was the highest at the initial phase of treatment, i.e., at the time of the operation, and thereafter was maintained at a low level without any significant change. Analysis of the ratio of monthly OOP costs to the total OOP expenditure amount during the two-year treatment period also showed that the first three months accounted for 25% of the entire OOP payment. Previous studies that analyzed trends in the incurrence of cancer treatment costs showed that a large amount of costs were incurred in the initial phase after diagnosis, and costs incurred in continuing treatment phases were much smaller (Brown et al., 2002; Yabroff et al., 2007; Riley et al., 1995; Kim et al., 2009; Yabroff et al., 2008). This study confirmed that not only the total costs, but the actual payments made by patients were concentrated in the initial stage of treatment. 

During the continuing phase from three months after the operation, the OOP payment ratio tended to decrease by time (year), but at the time of the operation the OOP payment ratio barely showed a difference depending on year. In other words, the OOP payment ratio at the time of the operation did not change when it came to recent years. NHI’s policy to largely reduce the patient copayment ratios for insured items and extend the range of insured items showed distinct effects in the continuing phase, but not in the initial phase. Because many uninsured treatments related to examination and operations are concentrated in the operation period, the financial burden on patients in the initial phase did not decrease significantly despite NHI’s efforts to alleviate it. This is also supported by the results of the analysis of OOP medical expenditure by statutory copayment expenses and costs of uninsured services, which demonstrated that in case of copayment costs, the level of expenditure concentration at the initial phase around the time of the operation showed almost no fluctuation by time, but in cases of uninsured costs, the level of concentration of expenditures in the initial phase increased.

NHI’s policies to reduce the financial burden on cancer patients did lower patient expenses in general, but it can be inferred that the high share of uninsured items among expenditures and the insufficient control of uninsured treatments reduced the effectiveness of the policy. The problem of the high concentration of patient burden in the initial phase of treatment (during the operation period) in comparison to the rest of the treatment period was especially evident after the implementation of this policy. Despite reduction in the copayment ratio and increased number of insured items for cancer patients, patients themselves may not have felt much of an effect in terms of alleviation of financial burden. In fact, even after the implementation of NHI’s policy, a significant number of cancer patients still felt that the burden of medical expenditure was too high (Kim et al., 2012). 

Aside from the insurer’s efforts to decrease the overall OOP payment ratio, there is also a need to smooth the medical expenditure or burden level by each period through managing patient expenditure time. In case of breast cancer patients, a large part of medical expenses incur in the initial phase around the operation date, whereas the cost burden in the continuing phase is relatively low, so alternatives measures such as flexible adjustment of the ratio of OOP expenditure amounts according to different treatment periods should be considered. For example, OOP payment for essential operations or treatments after diagnosis can be temporarily deferred for a set time period or be vastly decreased compared to the following period. Many countries implement an OOP maximum/limit system in order to reduce the burden of medical expenses (Barnieh et al., 2014; Dixon et al., 2017). South Korea’s NHI has also set different upper limits according to income level, and returns expenditures surpassing the limit to patients. However, this is managed on a yearly total amount basis, and not based on individual periods. Moreover, this only includes OOP payments for insured items; uninsured items are not covered by this policy regardless of the costs, which makes it less effective in terms of alleviating the payment burden on patients with serious diseases such as cancer, which incurs a large amount of uninsured costs.

This study has several limitations in its subjects and the analysis, and these limitations require caution when interpreting the results. This study used medical bills, but because medical bills do not include information that helps to determine the time of breast cancer diagnosis, incurrence of medical expenditures was analyzed based on the time of mastectomy. There is a possibility that the average medical expenditures were overestimated, considering that cases where surgery was impossible were excluded and cases where the diagnosed patient had chemotherapy first before the mastectomy were included. Second, cancer patient medical expenditures can vary depending on the treatment method according to the stage of disease, but because medical bills did not include this type of information, this study was not able to take this into account. Third, because this study analyzed the OOP expenditure trends among breast cancer patients, its results cannot be generalized to patients with different types of cancer. Fourth, because this study analyzed medical expenses directly related to cancer treatment, it did not take into account the indirect expenses such as transportation costs or payment for caregivers, and also cases where patients used other medical institutions during the study period. 

This is the first study to our knowledge that analyzed patient OOP expenditure trends by short time periods using actual medical bills from hospitals to calculate monthly OOP costs during the treatment period. According to its results, OOP payment burden on patients was concentrated in the initial phase of treatment around the time of operation, and items not covered by the NHI caused an additional increase in patient financial burden in the initial phase. In order to maximize effectiveness of policies that aim to alleviate cancer patients’ burden of medical expenses, there should be an individual and flexible approach to the issue that considers not only the average OOP ratio, but also the time expenditures are incurred and the possible burden on patients that new medical technology might bring.
